# To Predict the Requirement of Pharmacotherapy by OGTT Glucose Levels in Women with GDM Classified by the IADPSG Criteria

**DOI:** 10.1155/2018/3243754

**Published:** 2018-05-08

**Authors:** Gülen Yerlikaya, Veronica Falcone, Tina Stopp, Martina Mittlböck, Andrea Tura, Peter Husslein, Wolfgang Eppel, Christian S. Göbl

**Affiliations:** ^1^Department of Obstetrics and Gynecology, Division of Obstetrics and Feto-Naternal Medicine, Medical University of Vienna, Vienna, Austria; ^2^Center of Medical Statistics, Informatics, and Intelligent Systems, Section for Clinical Biometrics, Medical University of Vienna, Vienna, Austria; ^3^Metabolic Unit, Institute of Neuroscience, National Research Council, Padova, Italy

## Abstract

The aim of this study was to assess the association between OGTT glucose levels and requirement of pharmacotherapy in GDM patients classified by the IADPSG criteria. This study included 203 GDM patients (108 managed with lifestyle modification and 95 requiring pharmacotherapy). Clinical risk factors and OGTT glucose concentrations at 0 (G0), 60 (G60), and 120 min (G120) were collected. OGTT glucose levels were significantly associated with the later requirement of pharmacotherapy (ROC-AUC: 71.1, 95% CI: 63.8–78.3). Also, the combination of clinical risk factors (age, BMI, parity, and pharmacotherapy in previous gestation) showed an acceptable predictive accuracy (ROC-AUC: 72.1, 95% CI: 65.0–79.2), which was further improved when glycemic parameters were added (ROC-AUC: 77.5, 95% CI: 71.5–83.9). Random forest analysis revealed the highest variable importance for G0, G60, and age. OGTT glucose measures in addition to clinical risk factors showed promising properties for risk stratification in GDM patients classified by the recently established IADPSG criteria.

## 1. Introduction

The International Associations for Diabetes in Pregnancy Study Groups (IADPSG) guidelines for gestational diabetes mellitus (GDM) classification recommends a primary testing until the early third trimester by a 2 h-75 g oral glucose tolerance test (OGTT) [1, 2]. However, it was found that these recommendations resulted in a markedly increased number of GDM cases [3] containing a heterogenic group of pregnant women on the wide range of disturbed glucose metabolism. Therefore, most patients achieve adequate glycemic control with lifestyle modification while others need intensified pharmacologic therapy.

An early stratification into low- and high-risk patients could markedly improve the efficiency of treatment. Within the last years, different risk factors were identified which are associated with the requirement of glucose-lowering medications in women with GDM. These include age, prepregnancy BMI, history of GDM in previous pregnancy, and others [4–7]. Although previous studies indicated that increased OGTT glucose concentrations were related to adverse gestational and fetal outcome [8, 9], their specific performance for the prediction of pharmacotherapy in patients meeting the IADPSG criteria is less well investigated.

Therefore, this study aims to examine the ability of OGTT glucose levels to distinguish between low- (achieving glycemic targets with lifestyle modification) and high-risk (requiring pharmacotherapy) GDM patients diagnosed by the IADPSG criteria. Moreover, their contribution to discriminate low- and high-risk patients additional to more common and routinely documented risk factors should be assessed.

## 2. Materials and Methods

In this retrospective case-control study, we included 203 pregnant women diagnosed with GDM (108 managed with lifestyle modification and 95 requiring pharmacotherapy with insulin (*n* = 80), metformin (*n* = 11), or both (*n* = 4)), who attended our outpatient clinic between May 2015 and January 2017. GDM was diagnosed via universal testing by a 75 g-OGTT at 24–28 weeks of gestation, according to the IADPSG recommendations [1, 2]. Intensified lifestyle modification (ILS) including medical nutrition therapy was used as the first line intervention. All patients were instructed for capillary blood glucose monitoring and informed about glycemic treatment targets. Follow-ups in two to three weeks' time were scheduled, and blood glucose levels were reviewed during each appointment. The pharmacologic intervention was started at any time point when blood glucose targets were not achieved (i.e., <95 mg/dl at fasting or <140 mg/dl one hour after each meal) according to the current guidelines [10]. The following variables were collected from the medical records: age,gravidity, parity, prepregnancy BMI, history of GDM, first-degree relative with diabetes, and OGTT results at 0 min (G0), 60 min (G60), and 120 min (G120). Patients with preexisting diabetes as well as multiple pregnancies or those with missing OGTT glucose values were excluded. The study was approved by the Ethics Committee of the Medical University of Vienna and performed in accordance with the Declaration of Helsinki.

### 2.1. Statistical Analysis

Continuous and categorical variables were summarized as the means ± SD or median (IQR) and counts and percentages and compared by Student's *t*-test (or the Wilcoxon rank sum test for skewed distributed variables) and Pearson's chi-square test. Binary logistic regression was used to assess the probability of initiation of pharmacotherapy by OGTT glucose levels and risk factors such as age, prepregnancy BMI, parity, and history of GDM and others. Thereby, the predictive accuracy of the models was expressed as the area under the receiver operating characteristic curves (ROC-AUC). Unbiased recursive partitioning was performed, whereby decision trees were created by the conditional inference (Ctree) framework [11]. Thereby, random decision forests with ntree = 5000 were created to derive measures of variable importance (i.e., the average difference in predictive accuracy before and after permutation of a predictor variable over all 5000 trees). As supporting time-to-event analysis, the competing risk model of Fine and Gray [12] was conducted where preterm delivery (before 38 + 0 weeks of gestation) is considered a competing event to the start of pharmacotherapy. As single observations were missing at random for some covariables (G0: 1.5%; G60: 7.4%; G120: 8.4%; BMIPK: 1.0%; and history of GDM: 10.8%), multivariate imputations by chained equations were performed to estimate missing observations by the median value of 50 complete data sets. These imputations were used in logistic and proportional hazard regression models in order to achieve a maximum of information from our data. Group-specific comparisons of raw data are provided in Table 1. Statistical analysis was performed with R (version 3.4.1) and contributed packages (“mice” for data imputations as well as “pROC,” “party,” and “cmprsk” for data analysis) [13]. A two-sided *p* value ≤ 0.05 was considered statistically significant. *p* values were interpreted in an explorative manner aiming to generate new hypotheses, and no adjustment for multiple statistical testing was used if not otherwise indicated.

## 3. Results

**Figure 1:**
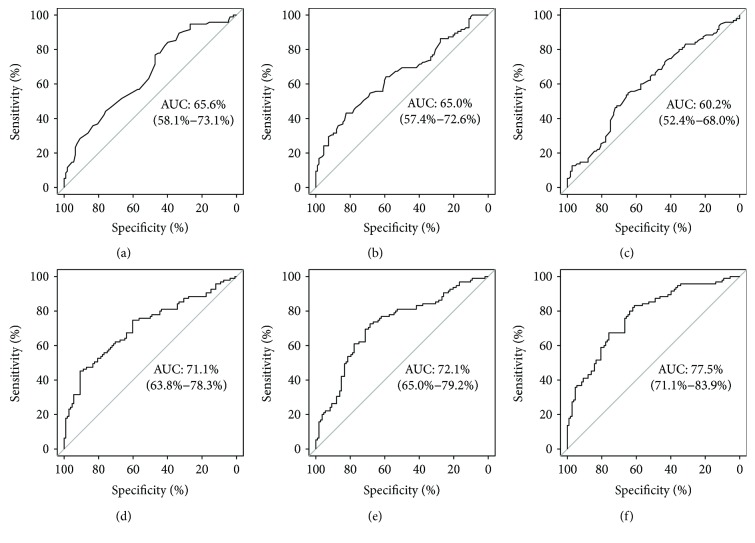
ROC curves for prediction of initiation of pharmacotherapy by OGTT glucose measurements at fasting (a), 60′ (b), and 120′ (c) after oral glucose load; combined information of OGTT glucose measurements (d); clinical predictors (age, pregestational BMI, parity, history of GDM, family history with type 2 diabetes, and time of diagnosis) (e); and OGTT glucose measurements and clinical predictors (f).

**Figure 2:**
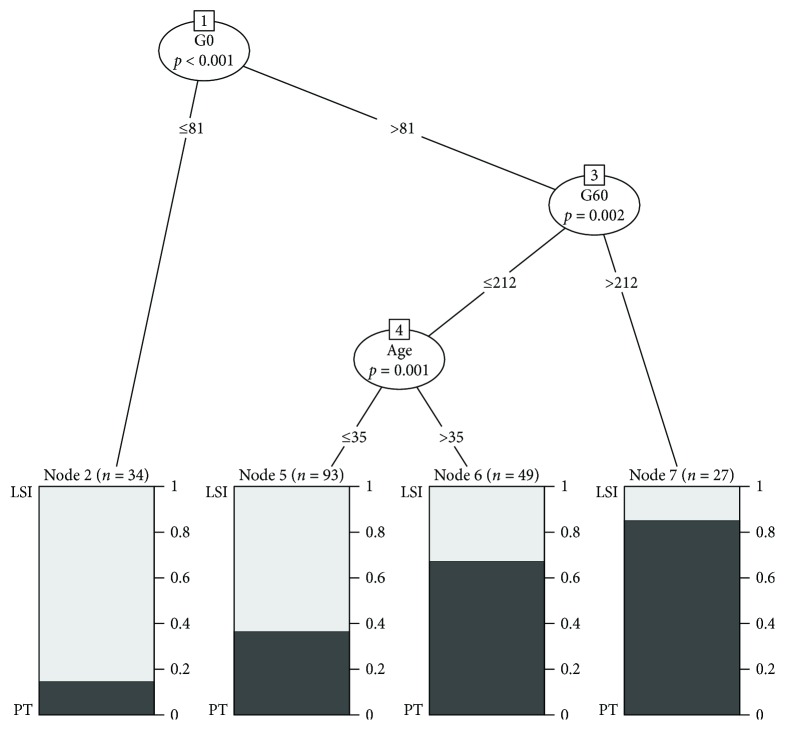
Example of a conditional inference tree for the prediction of intensified pharmacologic intervention in GDM patients. Bonferroni adjusted *p* values are given for each inner node, and the fraction of lifestyle intervention (LSI) and initiation of pharmacotherapy (PT) is provided at each terminal node.

A descriptive comparison of the data is provided in Table 1. It was found that mothers requiring pharmacotherapy were older, had higher BMI, and reached higher plasma glucose concentrations during the OGTT. Although univariable analysis revealed significant associations between plasma glucose levels and risk for pharmacologic intervention, the relative contribution of each glucose measure including their mean value was only modest to fair (ROC-AUC ranged between 60.2 and 65.6% as provided in Figure 1(a)–1(c)). The predictive performance was, however, markedly improved when the information of all three OGTT glucose measurements was combined by multiple logistic regression (model 1: ROC-AUC: 71.1, Figure 1(d)). Also, the combination of clinical variables (age, pregestational BMI, parity, history of GDM in previous gestation, family history with type 2 diabetes, and time of diagnosis) showed an acceptable discrimination between low- and high-risk patients (model 2: ROC-AUC: 72.1, Figure 1(e); *p* = 0.834 versus model 1), which was further improved when OGTT glucose levels were included as additional predictors (model 3: ROC-AUC: 77.5, Figure 1(f); *p* = 0.030 versus model 1 and *p* = 0.033 versus model 2). Random forest analysis revealed the highest variable importance for G0 (1.11 × 10^−2^), G60 (0.96 × 10^−2^), and age (0.80 × 10^−2^), while the importance of the remaining predictors was shown to be inferior (G120: 0.24 × 10^−2^, BMI: 0.40 × 10^−2^, parity: 0.05 × 10^−2^, history of GDM: 0.31 × 10^−2^, family history with type 2 diabetes: −0.17 × 10^−2^, and time of diagnosis: 0.13 × 10^−2^). An exemplary decision tree with possible cut-off values for G0, G60, and age is also provided. A very low risk for pharmacotherapy was observed for patients with G0 ≤ 81 mg/dl, whereas patients with G0 > 81 and G60 > 212 had a very high risk for pharmacologic intervention (Figure 2). Pharmacotherapy was initiated at a median time of 31.1 weeks of gestation. G0 (HR: 1.12, 95% CI: 1.01–1.24, for an increase of 10 mg/dl), G60 (HR: 1.14, 95% CI: 1.05–1.24, for an increase of 10 mg/dl), and age (HR: 1.25, 95% CI: 1.04–1.51, for an increase of 5 years) were also found to be independent predictors for pharmacologic intervention by time-to-event analysis, accounting for preterm delivery (before 38 + 0 weeks). Moreover, the combined information of G0, G60, and age showed a fair discrimination when included in a logistic regression model to predict the probability for delivering LGA offspring (ROC-AUC: 64.7%, 95% CI: 54.1–75.3). However, only G0 reached a significance in this model (*p* = 0.012).

## 4. Discussion

This study assessed the association between glucose measures derived from a diagnostic 75 g-OGTT and future requirement of pharmacotherapy in women classified with GDM according to the IADPSG recommendations. Albeit the individual glucose measurements (G0, G60, and G120) were significantly associated with the risk of requiring glucose-lowering medication, their predictive performance was markedly improved when the information of all three OGTT glucose values was combined by multivariable logistic regression. The model discrimination of clinical risk factors was also improved when OGTT glucose measurements were added as further predictors. Thereby, G0 followed by G60 and age achieved the highest variable importance measures.

These findings are of clinical relevance: in 2010, the IADPSG recommended novel criteria for GDM classification based on one-step OGTT testing in all pregnant women [1]. These criteria were more recently adopted by the WHO and several local health care organizations [2]. However, it was criticized that this novel established strategy would substantially increase the number of GDM patients by additionally identifying very subtle alterations in disturbed glucose metabolism [14]. This limitation of the IADPSG approach emphasizes the need for accurate risk stratification early after diagnosis to separate low- from high-risk pregnancies, that is, patients who achieve adequate glycemic control with lifestyle modification and those who require more intensified treatment strategies. Although already previous research assessed a constellation of risk factors associated with antenatal insulin therapy, most of the available studies used earlier diagnostic algorithms (mostly two-step approaches) and therefore contain study populations with different risk profiles as compared to patients with GDM identified by the one-step IADPSG approach. While in accordance to our findings some of these previous studies also indicated a significant association between elevated fasting glucose levels and requirement of pharmacotherapy [4–7], more controversial results are reported for elevated post load OGTT glucose: Wong and Jalaludin found that G120 in addition to G0, BMI at booking, and gestational week at diagnosis was independently associated with insulin therapy [6], whereas in another study, which used a comparable diagnostic approach (two-step screening according to the Australasian Diabetes in Pregnancy Society (ADIPS)), the predictive performance of G120 lost its significance in multivariable regression [4]. A further Australian study identified several clinical determinants of antenatal insulin treatment including G0, G60, and HbA1c [5]. Nevertheless, these variables explained only a small amount of the variance and in accordance with others the authors concluded that antenatal factors including glycemic measures were insufficient to predict the attributable risk for intensified pharmacotherapy [5, 15]. This is in contrast to our finding in GDM patients identified by the one-step IADPSG approach, where aggregated information of glycemic as well as clinical risk factors showed an acceptable accuracy with an ROC-AUC ranging from 71.1% (glycemic parameters) to 77.5% (glycemic parameters and clinical risk factors). Only two further studies were identified using the IADPSG criteria for GDM classification: Bakiner et al. found that G0 (ROC-AUC: 73.4%) and HbA1c (ROC-AUC: 67.7%) were independently associated with antenatal insulin treatment in a retrospective study on 300 pregnancies [16]. However, dynamic OGTT glucose values were not reported in this study. Mitra et al. observed a significant association between G60 and antenatal insulin treatment with an ROC-AUC of 83.1% [17]. However, the sample size of this study was very small with 8 out of 83 patients requiring pharmacotherapy.

The importance of dynamic glucose measurements for risk stratification is also supported by previous research in nonpregnant patients indicating strong associations between elevated fasting and dynamic OGTT glucose measures with decreased insulin sensitivity and *β*-cell dysfunction [18]. Accordingly, a higher degree of insulin resistance and impaired insulin secretion was recently found to characterize patients with GDM requiring pharmacotherapy [19]. A standardized examination of HbA1c at the time of diagnosis was not available in our study, which could be seen as a limitation of this work. However, in contrast to dynamic measures of glucose levels, HbA1c is a weak surrogate of insulin sensitivity or secretion and gives almost no additive information to glycemic parameters assessed during the OGTT [20]. HbA1c is further subjected to pregnancy-specific changes [21, 22]. Although we provided an exemplary decision tree including the main predictors for intensified therapy identified by our study, this algorithm needs further validation. Of note, we were not able to assess lifestyle habits or several components of the “metabolic syndrome” due to the retrospective nature of this work. This data could be of additional importance and needs to be addressed in future prospective studies.

## 5. Conclusions

In summary, we found that fasting and dynamic OGTT glucose measures in addition to clinical risk factors showed promising properties for risk stratification in GDM patients classified by the recently established IADPSG criteria. These findings should be considered in future studies to establish an accurate separation for the early treatment of high-risk patients.

## Figures and Tables

**Table 1:** Characteristics of the study population.

	*n*	GDM-LSI (*n* = 108)	*n*	GDM-PT (*n* = 95)	*p* value
Age (years)	108	31.5 ± 5.9	95	34.6 ± 5.9	<0.001
BMI (kg/m^2^)	107	27.8 ± 6.4	94	30.1 ± 6.9	0.014
Fertility treatment	108	6 (5.6%)	95	8 (8.4%)	0.421
Gravidity (count)	108	3.0 (2.0–4.0)	95	3.0 (2.0–5.0)	0.029
Gravidity (>1)	108	87 (80.6%)	95	83 (87.4%)	0.189
Parity (count)	108	1.0 (0.0–2.0)	95	1.0 (1.0–2.5)	0.054
Parity (≥1)	108	73 (67.6%)	95	74 (77.9%)	0.101
pGDM	102	19 (18.6%)	87	34 (39.0%)	0.002
G0 (mg/dl)	105	90.0 ± 10.8	95	98.8 ± 19.2	<0.001
G60 (mg/dl)	101	173.2 ± 29.5	87	189.0 ± 30.8	<0.001
G120 (mg/dl)	98	129.7 ± 29.0	88	140.4 ± 33.9	0.021
Gmean (mg/dl)	98	130.6 ± 15.2	87	141.7 ± 20.3	<0.001
Week diagnosis	108	28.0 (26.4–30.3)	95	27.7 (25.6–29.4)	0.032
Week treatment start		—	95	31.1 (28.0–33.3)	
BW (pct)	108	50.5 (24.0–70.5)	95	50.0 (26.0–86.0)	0.427
LGA	108	12 (11.1%)	95	21 (22.1%)	0.034

Data are mean ± standard deviations or median and interquartile range (IQR) for pregnant women affected by GDM and treated with lifestyle modification (GDM-LSI) or requiring pharmacologic treatment (GDM-PT). BMI: pregestational body mass index; pGDM: previous pregnancy with gestational diabetes mellitus; G0: fasting plasma glucose; G60: plasma glucose 60 min after oral glucose load; G120: plasma glucose 120 min after oral glucose load; BW: birth weight; LGA: large for gestational age offspring.

## Data Availability

Data are available from the corresponding author for researchers who meet the criteria for access to confidential data. Please contact Christian Göbl (email: christian.goebl@meduniwien.ac.at).
